# Clinical and Laboratory Factors in Predicting Mortality Among COVID-19 RT-PCR Positive Patients: A Retrospective Observational Study From a Tertiary Care Center

**DOI:** 10.7759/cureus.19791

**Published:** 2021-11-21

**Authors:** Raja Sundaramurthy, Suryakumar Balasubramanian, Vithiya Ganesan, Pearl Aggarwal, Tarun Parvataneni, Devi Parvathy Jyothi Ramachandran Nair, Raja Prahadeesh Saravanan

**Affiliations:** 1 Microbiology, All India Institute of Medical Sciences, Bibinagar, IND; 2 Critical Care Medicine, Velammal Medical College Hospital & Research Institute, Madurai, IND; 3 Microbiology, Velammal Medical College Hospital & Research Institute, Madurai, IND; 4 Internal Medicine, Golden Hospital, Zirakpur, IND; 5 Internal Medicine, Siddavanahalli Nijalingappa Medical College and HSK Hospital and Research Centre, Bagalkot, IND; 6 Internal Medicine, Tirunelveli Medical College, Tirunelveli, IND; 7 Internal Medicine, R.R Hospital, Tuticorin, IND

**Keywords:** co-morbidities, coronavirus disease (covid-19), clinical and laboratory characteristics, acute respiratory distress syndrome [ards], mortality predictors

## Abstract

Background: In coronavirus disease 2019 (COVID-19) patients, risk stratification based on clinical presentation, co-morbid illness, and combined laboratory parameters is essential to provide an adequate, timely intervention based on an individual’s conditions to prevent mortality among cases.

Methods: A retrospective observational study was carried out from June to October 2020, including all reverse transcription-polymerase chain reaction (RT-PCR) positive COVID-19 non-survivors and control group survivors randomly selected after age and sex matching. Clinical and demographic information was collected from the medical records. Categorical variables were expressed by frequency and percentage. To explore the risk factors associated with mortality, univariable and multivariable logistic regression models were used.

Results and discussions: All non-survivors (n = 100) and 100 survivors (out of 1,018) were analyzed. Male gender (67.4%) was the independent risk factor for COVID-19 infection. Advanced age group, diabetes, cardiovascular, neurological, and hypertensive co-morbidities were statistically associated with mortality. Cardiac arrest and acute kidney injury (AKI) were the most common complications. Mortality is significantly associated with lymphopenia and raised lactate dehydrogenase (LDH), as shown by higher odds. In addition, raised neutrophils, monocytes, aspartate aminotransferase (AST), serum creatinine, interleukin 6 (IL-6), and C-reactive protein (CRP) are also significantly associated with mortality. The most common causes of death were respiratory failure (84%) and acute respiratory distress syndrome (77%). Of the non-survivors, 92% received corticosteroids, 63% were on high-flow nasal cannula oxygen therapy, 29% were mechanically ventilated, and 29% received tocilizumab.

Conclusion: Serial monitoring of neutrophils, lymphocytes, D-dimer, procalcitonin, AST, LDH, CRP, IL-6, serum creatinine, and albumin might provide a reliable and convenient method for classifying and predicting the severity and outcomes of patients with COVID-19.

## Introduction

The coronavirus disease 2019 (COVID-19), which is caused by severe acute respiratory syndrome coronavirus 2 (SARS-CoV-2), originated in December 2019 in Wuhan, China. It has ever since rapidly spread worldwide causing morbidity and mortality in its way. In March 2020, the World Health Organization (WHO) declared the COVID-19 outbreak as a pandemic [1]. Though COVID-19 primarily affects the respiratory system, the clinical presentation of COVID-19 shows significant heterogeneity, ranging from asymptomatic, mild, moderate, and severe disease with multi-organ dysfunction leading to death. Therefore, risk stratification is essential to formulate better treatment plans, either to admit in hospital or community isolation or home quarantine. The most severe cases of COVID-19 are often due to respiratory failure, which often requires mechanical ventilation. This in turn leads to a higher mortality rate. In a recent study, it was seen that the mortality was 40.8% among patients receiving invasive mechanical ventilation, 39% among patients receiving extracorporeal membrane oxygenation (ECMO), and 71.6% for patients on invasive mechanical ventilation, vasoactive drugs, and new renal replacement therapy [2]. The fatality rate was very high among the severe or critically ill patients (49%) when compared to the overall (2.3%) case fatality as per the epidemiological data from the Centers for Disease Control and Prevention [3]. Some studies have reported that among COVID-19 patients, the older age group with comorbidities has been associated with a poor prognosis [4].

The risk/prognosis/mortality predictor based on clinical presentation, co-morbid illness, combined laboratory parameters (biochemical, hematological, and inflammatory markers), and imaging will play a significant role in understanding the disease and providing an adequate, timely intervention based on the individual's conditions [5]. Due to the heterogeneous nature of COVID-19, our study was planned to analyze clinical presentation with comorbidities and laboratory factors in predicting mortality among COVID-19 reverse transcription-polymerase chain reaction (RT-PCR) positive patients.

## Materials and methods

This retrospective observational study was carried out in our tertiary care center after obtaining institutional ethical committee clearance. The study included adult patients (>18 years) admitted into the COVID-19 isolation unit of the hospital with positive RT-PCR who died due to COVID-19 between June and October 2020. During the same period, patients admitted with COVID-19 with positive RT-PCR who were discharged/recovered (survivors) were randomly selected and included as the control group after age and sex matching.

Information regarding demographic details, clinical presentations, co-morbid conditions, and biochemical (renal, liver function tests, lactate dehydrogenase, D-dimer, interleukin 6 [IL-6], serum ferritin, fibrinogen), hematological, including coagulation profile, and inflammatory markers (C-reactive protein [CRP], procalcitonin) along with treatment details and duration of stay in the hospital for both groups were collected from the medical records using a standard data collection form, which was verified by other researchers for any difference.

Definitions

Confirmed cases of COVID-19, acute respiratory distress syndrome (ARDS), sepsis, and septic shock are defined by the Revised Guidelines on Clinical Management of COVID-19, Government of India [6]. Acute kidney injury was diagnosed according to the Kidney Disease Improving Global Outcomes (KDIGO) clinical practice guidelines [7].

Statistical analysis

Our study is an age and sex-matched case-control study with 100 cases and 100 controls. Data analyses were done using STATA software, version 16 (StataCorp LLC, College Station, Texas). The descriptive results for the categorical variables were displayed by frequency and percentage. For continuous variables, median and ranges were used. To explore the risk factors associated with in-hospital death, univariable and multivariable logistic regression models were used.

As this was a matched case-control study using matching individuals, conditional logistic regression was used to estimate the association between the predictors of interest with survivors and non-survivors of COVID-19 within each matched set of cases and controls, and the level of significance was set at a two-tailed p-value of less than 0.05.

## Results

A total of 1,118 patients were admitted into the COVID-19 isolation unit of our hospital during the study period. Statistically, a significant difference was found in the age group of patients (p < 0.0001), and no difference was found in gender analysis (p = 0.0686) among survivors and non-survivors (Table 1).

**Table 1:** Profile of non-survivors and survivors admitted in the COVID-19 isolation unit of our tertiary care institute.

Variable		Non-survivors (N = 100)	Survivors (N = 1,018)	p-value
Gender	Female	25 (25)	340 (33.4)	0.0686
	Male	75 (74)	678 (66.6)	
Age	Mean	64.11	52.53	<0.0001
	Median	65.00	55.00	
	Mode	62	55	
	Std. deviation	10.909	16.145	
	Minimum	35	3	
	Maximum	86	87	

For our study purpose, we have included all non-survivors (n = 100) and we randomly selected 100 patients from the survivors for analysis after age and sex matching. Analysis of clinical presentations and comorbid conditions among non-survivors and survivors are presented in Tables 2, 3, respectively. There was a statistically significant difference in the clinical presentation of shortness of breath among the cases and controls. Other clinical manifestations did not show any statistical significance. Diabetes, hypertension, cardiovascular, and neurological risk factors were more associated with non-survivors and were statistically significant.

**Table 2:** Analysis of clinical presentation among non-survivors and survivors.

	Total (%)	Non-survivors (n = 100)	Mean number of days in non-survivors	Survivors (n = 100)	Mean number of days in survivors	p-value
Fever	118 (59)	67	4.95	51	5.12	0.878
Myalgia	69 (34)	42	4.47	27	5	0.738
Sore throat	15 (7.5)	11	3.9	4	3.5	0.703
Cough	82 (41)	38	5.33	44	5.67	0.85
Nasal congestion	7 (3.5)	4	5.75	3	3.33	0.09
Shortness of breath	93 (46.5)	64	4.45	29	3.44	0.031
Vomiting	14 (7)	7	3	7	4.42	0.827
Diarrhea	15 (7.5)	8	4.37	7	2.42	0.132

**Table 3:** Analysis of comorbidities among non-survivors and survivors.

Risk factor		Non-survivors (n%)	Survivors (n%)	p-value
Diabetes	No	41 (41.0)	56 (56.0)	0.024
	Yes	59 (59.0)	44 (44.0)	
Hypertension	No	50 (50.0)	63 (63.0)	0.043
	Yes	50 (50.0)	37 (37.0)	
Cardiovascular	No	79 (79.0)	89 (89.0)	0.041
	Yes	21 (21.0)	11 (11.0)	
Pulmonary	No	95 (95.0)	96 (96.0)	0.500
	Yes	5 (5.0)	4 (4.0)	
Renal	No	95 (95.0)	96 (96.0)	0.500
	Yes	5 (5.0)	4 (4.0)	
Neurological	No	87 (87.0)	95 (95.0)	0.041
	Yes	13 (13.0)	5 (5.0)	
Cancer	No	98 (98.0)	98 (98.0)	0.689
	Yes	2 (2.0)	2 (2.0)	

**Figure 1:**
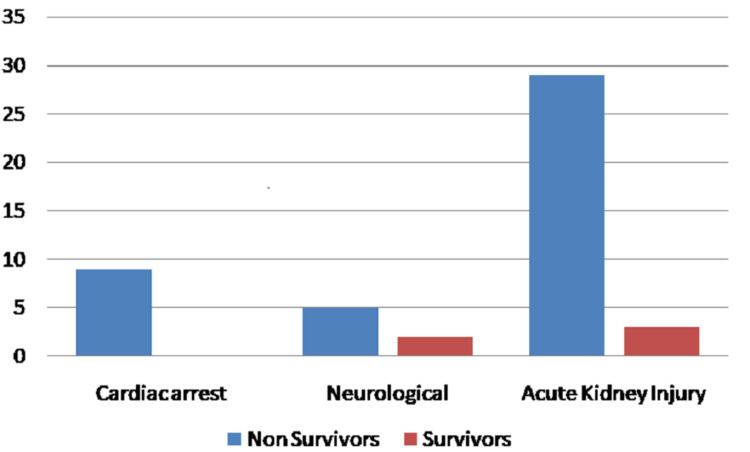
Complications among non-survivors and survivors.

Analysis of complications among non-survivors and survivors showed cardiac arrest (p = 0.0022) and acute kidney injury (p < 0.0001) were more with the non-survivors (Figure 1).

Analysis of complete blood count and coagulation profile among non-survivors and survivors is given in Table 4. There was a statistically significant difference in the total count, polymorphs, lymphocytes, and monocytes among the cases and controls. Also, absolute neutrophil and absolute lymphocyte count showed a statistically significant difference between non-survivors and survivors. No statistical difference was found in the analysis of the coagulation profile of prothrombin time (PT) and activated partial thromboplastin time (APTT).

**Table 4:** Analysis of complete blood count and coagulation profile among non-survivors and survivors. PT: prothrombin time; APTT: activated partial thromboplastin time; sec: seconds; cu.mm: cubic millimeters; mg/dL: milligram per deciliter.

Variables		Non-survivors (n = 100)	Survivors (n = 100)	p-value
Polymorphs (cells/cu.mm)	1,500-8,000	48 (51.6)	67 (74.4)	0.001
	>8,000	45 (48.4)	23 (25.6)	
Lymphocytes (cells/cu.mm)	1,000-4,800	35 (37.6)	65 (72.2)	0.000
	<1,000	58 (62.4)	25 (27.8)	
Monocytes (cells/cu.mm)	285-500	83 (89.2)	69 (76.7)	0.019
	>500	10 (10.8)	21 (23.3)	
Eosinophils (cells/cu.mm)	0-500	92 (98.9)	88 (97.8)	0.488
	>500	1 (1.1)	2 (2.2)	
Basophils (cells/cu.mm)	0-300	92 (98.9)	90 (100.0)	0.508
	>300	1 (1.1)	0 (0.0)	
Hemoglobin (mg/dl)	12-17	86 (92.5)	84 (93.3)	0.525
	<12	7 (7.5)	6 (6.7)	
Platelets (cells/cu.mm)	1.5-4 lakh	78 (83.9)	82 (91.1)	0.104
	<1.5 lakh	15 (16.1)	8 (8.9)	
PT (sec)	11-13	18 (37.5)	16 (50.0)	0.190
	>13	30 (62.5)	16 (50.0)	
APTT (sec)	30-35	45 (93.8)	30 (93.8)	0.670
	>35	3 (6.2)	2 (6.2)	

Analysis of enzymes, interleukins, sepsis markers, and other coagulation profiles among cases and controls is given in Table 5. There was a statistically significant difference in the blood urea, lactate dehydrogenase (LDH), aspartate aminotransferase (AST), IL-6, and quantitative CRP among the cases and controls. Other parameters did not show any statistical difference.

**Table 5:** Analysis of enzymes, interleukins, sepsis markers, and other coagulation profiles among non-survivors and survivors. LDH: lactate dehydrogenase; AST: aspartate aminotransferase; ALT: alanine aminotransferase; IL-6: interleukin 6; CRP: C-reactive protein; mg/dL: milligram per deciliter; ng/dL: nanogram per deciliter; g/dL: gram per deciliter; ng/mL: nanogram per milliliter; pg/mL: picogram per milliliter; mg/L: milligram per liter.

Variables		Non-survivors (n = 100)	Survivors (n = 100)	p-value
Blood urea (mg/dl)	≤50	59 (63.4)	68 (78.2)	0.022
	>50	34 (36.6)	19 (21.8)	
Creatinine (mg/dl)	≤1.5	78 (83.9)	77 (89.5)	0.187
	>1.5	15 (16.1)	9 (10.5)	
LDH (mg/dl)	≤470	1 (1.3)	10 (13.3)	0.004
	>470	76 (98.7)	65 (86.7)	
Albumin (g/dl)	>2.5	85 (98.8)	82 (98.8)	0.743
	≤2.5	1 (1.2)	1 (1.2)	
AST (g/dl)	≤40	19 (22.1)	41 (49.4)	0.000
	>40	67 (77.9)	42 (50.6)	
ALT (g/dl)	≤40	50 (58.1)	57 (68.7)	0.104
	>40	36 (41.9)	26 (31.3)	
D-dimer (ng/dl)	≤500	26 (40.0)	25 (46.3)	0.307
	>500	39 (60.0)	29 (53.7)	
Fibrinogen (mg/dl)	≤498	39 (70.9)	27 (69.2)	0.519
	>498	16 (29.1)	12 (30.8)	
Procalcitonin (ng/ml)	Low risk < 0.5	59 (79.7)	58 (87.9)	0.422
	Probable sepsis = 0.5-2	11 (14.9)	5 (7.6)	
	Moderate sepsis = 2-10	3 (4.1)	3 (4.5)	
	Severe sepsis > 10	1 (1.4)	0 (0.0)	
IL-6 (pg/ml)	≤7.5	0 (0.0)	3 (20.0)	0.040
	>7.5	27 (100.0)	12 (80.0)	
Quantitative CRP (mg/L)	≤10	2 (5.0)	6 (21.4)	0.047
	>10	38 (95.0)	22 (78.6)	

In univariable analysis, the odds of in-hospital mortality were higher in patients with diabetes, hypertension, renal disorder, and coronary heart disease. Neutrophilia, lymphopenia, monocytosis, elevated AST, LDH, and serum ferritin were also associated with increased mortality. In multivariate analysis, lymphopenia and elevated LDH were associated with death (Table 6).

**Table 6:** Univariate and multivariate analysis of risk factors associated with non-survivors. AST: aspartate aminotransferase; ALT: alanine aminotransferase; LDH: lactate dehydrogenase; IL-6: interleukin 6; CRP: C-reactive protein; cu.mm: cubic millimeters; g/dl: gram per deciliter; mg/dL: milligram per deciliter; pg/mL: picogram per milliliter; ng/mL: nanogram per milliliter; mg/L: milligram per liter.

Variables	Category	Total	Univariable OR	p-value	Multivariable OR	p-value
Polymorphs (cells/cu.mm)	1,500-8,000	115 (62.84)	1.0 (Ref)		1.0 (Ref)	
	>8,000	68 (37.16)	2.357	0.007	0.895	0.601
Lymphocytes (cells/cu.mm)	1,000-4,800	100 (54.64)	1.0 (Ref)		1.0 (Ref)	
	<1,000	82 (44.81)	3.417	0.000	3.737	0.036
	>4800	1 (0.55)	-	-		
Eosinophils (cells/cu.mm)	0-500	180 (98.36)	1.0 (Ref)			
	>500	3 (1.64)	0.5	0.571		
Basophils (cells/cu.mm)	0-300	182 (99.45)	1.0 (Ref)			
	>300	1 (0.55)	1.24e + 15	1.000		
Monocytes (cells/cu.mm)	285-500	152 (83.06)	1.0 (Ref)			
	>500	31 (16.94)	0.375	0.040		
Platelets (cells/cu.mm)	(1.5 lakhs to 4 lakhs)	160 (87.43)	1.0 (Ref)			
	(<1.5 lakh)	23 (12.57)	2.2	0.144		
Albumin (g/dl)	>2.5	167 (98.82)	-	-		
	≤2.5	2 (1.18)	-	-		
AST (g/dl)	≤40	60 (35.50)	1.0 (Ref)		1.0 (Ref)	
	>40	109 (64.50)	4.143	0.001	1.741	0.525
ALT (g/dl)	≤40	107 (63.31)	1.0 (Ref)		1.0 (Ref)	
	>40	62 (36.69)	1.846	0.075	0.506	0.303
Blood urea (mg/dl)	≤50	127 (70.56)	1.0 (Ref)		1.0 (Ref)	
	>50	53 (29.44)	1.833	0.091	0.797	0.752
Creatinine (mg/dl)	≤1.5	155 (86.59)	1.0 (Ref)			
	>1.5	24 (13.41)	1.714	0.257		
LDH (mg/dl)	≤470	11 (7.24)	1.0 (Ref)		1.0 (Ref)	
	>470	141 (92.76)	8	0.050	1.004	0.007
D-dimer (ng/ml)	≤500	51 (42.86)	1.0 (Ref)			
	>500	68 (57.14)	0.889	0.808		
Fibrinogen (mg/dl)	≤498	66 (70.21)	1.0 (Ref)			
	>498	28 (29.79)	2	0.571		
IL-6 (pg/ml)	≤7.5	3 (7.14)	-	-		
	>7.5	39 (92.86)	-	-		
Procalcitonin (ng/ml)	<0.5	117 (81.25)	1.0 (Ref)			
	0.5-2	19 (13.19)	1.5	0.530		
	2-10	7 (4.86)	2.00	0.423		
	>10	1 (0.69)	-	-		
Quantitative CRP (mg/L)	≤10	8 (11.76)	1.0 (Ref)			
	>10	60 (88.24)	3	0.341		
Prothrombin time (sec)	≤13	34 (42.50)	1.0 (Ref)			
	>13	46 (57.50)	1.167	0.782		
Activated partial thromboplastin time (sec)	≤35 >35	75 (93.75) 5(6.2)	1.0 (Ref)			
Ferritin (ng/ml)	≤250	39 (27.66)	1.0 (Ref)			
	>250	102 (72.34)	11	0.022		

Among non-survivors, biochemical and hematological markers taken at the time of admission and 24 hours before death showed progressive high total WBC count, neutrophil count, lymphocytopenia, low serum albumin, elevated ALT, blood urea, raised LDH, D-dimer, ferritin, and procalcitonin levels and are significantly associated with mortality (Table 7).

**Table 7:** Analysis of biochemical and hematological markers of non-survivors at time of admission and 24 hours before death. AST: aspartate aminotransferase; SGOT: serum glutamic oxaloacetic transaminase; ALT: alanine aminotransferase; SGPT: serum glutamic pyruvic transaminase; LDH: lactate dehydrogenase; IL-6: interleukin 6; ESR: erythrocyte sedimentation rate; cu.mm: cubic millimeters; g/dL: gram per deciliter; mg/dL: milligram per deciliter; ng/mL: nanogram per milliliter; pg/mL: picogram per milliliter; mm/hour: millimeters per hour.

	Non-survivors mean value at admission	Non-survivors mean value 24 hours before death	p-value
White blood cell count (cells/cu.mm)	10,925	18,157	<0.0001
Neutrophil count (cells/cu.mm)	9,422	16,608	<0.0001
Lymphocyte count (cells/cu.mm)	921	793	0.0115
Eosinophil count (cells/cu.mm)	21	46	0.096
Basophil count (cells/cu.mm)	18	25	0.176
Monocyte count (cells/cu.mm)	541	684	0.077
Hemoglobin (g/dl)	12.6	12.9	0.37
Platelet count (cells/cu.mm)	237,000	240,000	0.856
Albumin (g/dl)	3.69	3.18	<0.0001
AST (SGOT) (g/dl)	72	78.9	0.43
ALT (SGPT) (g/dl)	48.6	71.3	0.004
Blood urea (mg/dl)	48.21	97.89	<0.0001
Serum creatinine (mg/dl)	1.1	1.5	0.0086
LDH (mg/dl)	1,114	1,598	<0.0001
D-dimer (ng/ml)	1,983	7,108	<0.0001
Ferritin (ng/ml)	640	827	0.038
Fibrinogen (mg/dl)	436	427	0.741
IL-6 (pg/ml)	192.7	238.6	0.05
Procalcitonin (ng/ml)	0.75	3.62	0.0002
ESR (mm/hour)	30.7	33.5	0.159

**Figure 2:**
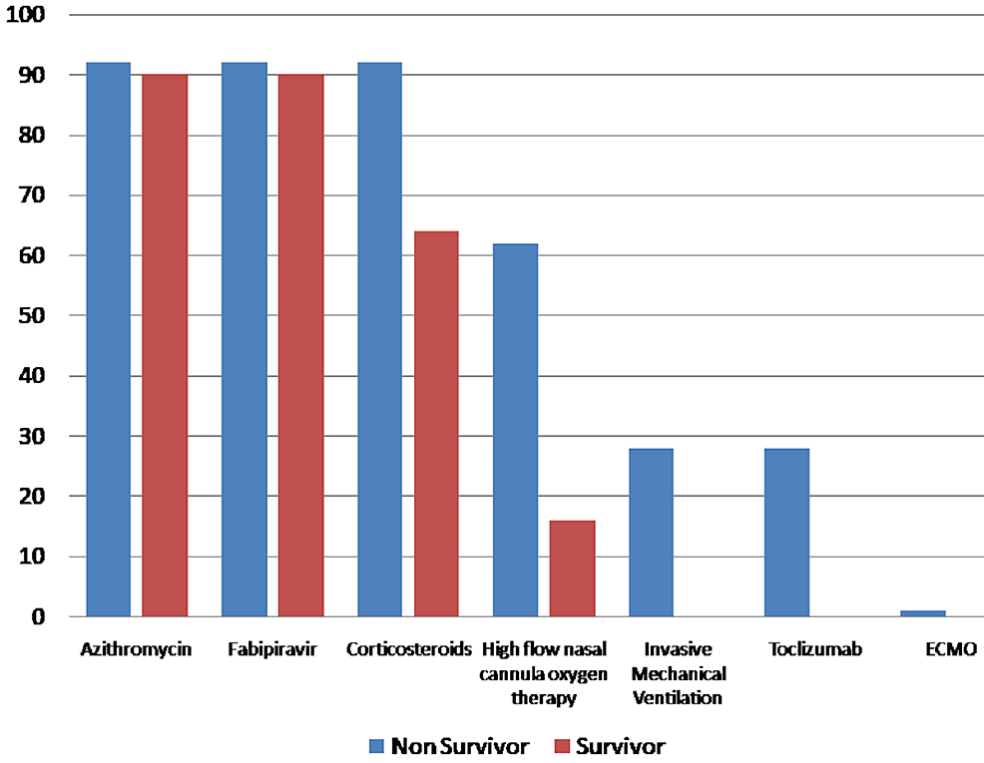
Treatment analysis among survivors and non-survivors. ECMO: extracorporeal membrane oxygenation.

The most common causes of death were respiratory failure (84%) and ARDS (77%), followed by cardiac arrest (10%) and septic shock (4%). Analysis of treatment given among non-survivors and survivors showed 92% of non-survivors got corticosteroids, 63% needed high-flow nasal cannula oxygen therapy, 29% had invasive mechanical ventilation, and 29% received tocilizumab with a statistically significant difference of p < 0.0001 (Figure 2).

## Discussion

Analysis of COVID-19 patients admitted to our tertiary care center revealed that the male gender preponderance in COVID-19 infection is 67.4% (754/1,118). This is in concordance with Bwire and Guan et al. [8,9]. This may be due to the sex hormones-driven immunological differences between males and females, and the higher gene expression of angiotensin-converting enzyme 2 (ACE2) in males. Men also tend to engage in more risky behaviors like ignoring frequent hand-washing, mask-wearing, and staying-at-home orders.

Also, our study reveals nearly 11.2% (76/678) mortality in males when compared to 7.1% (24/340) in females, but there is no statistically significant difference (p = 0.068) found in the gender analysis to mark male gender as an independent marker of mortality. The analysis of the age group reveals that advanced age by itself served as an independent marker of mortality with a p-value of <0.0001. Our finding is in concordance with Perrotta et al. [10]. This may be because of immunosenescence and the presence of co-morbid disorders in elderly individuals, which might promote a cytokine storm when infected with COVID-19 leading to life-threatening complications.

Analysis of clinical presentation reveals that fever (59%), shortness of breath (46.5%), cough (41%), and myalgia (34%) were the most common presenting symptoms in our setup. Of these, patients who presented with sudden onset of breathlessness were associated with increased mortality (p = 0.031). Co-morbid conditions analysis reveals that patients with diabetes (p = 0.024), cardiovascular diseases (p = 0.041), neurological diseases (p = 0.041), and hypertension (p = 0.043) had increased mortality. Our findings are in concordance with Singh et al. and Sanyaolu et al. [11,12]. Cardiovascular damage and acute kidney injury (AKI) were the significant complications that ended in mortality. Cardiac arrest was documented in nine (4.5%) patients among whom eight patients were above the age of 50 years and diabetic, two of them presented with diabetic ketoacidosis, three of them had pre-existing cardiovascular diseases, and one patient presented with acute myocardial infarction.

Despite the very limited information on kidney involvement in COVID-19, AKI is a well-documented complication. In a study by Zhou et al. [13], 50% of non-survivors and 1% of survivors developed AKI (p < 0.001). In our study, AKI was seen with 29% of non-survivors and 3% of survivors; that is AKI is significantly more in the non-survivor group than the survivor group (p < 0.001). AKI was diagnosed according to the KDIGO clinical practice guidelines by Khwaja et al. [7].

Though not statistically significant, we had seven patients (five fatal and two non-fatal) who presented with neurological complications. The symptoms on presentation included giddiness, seizures, acute limb ischemia, and weakness. Among the seven patients, four of them presented with acute infarct, two of them with subarachnoid hemorrhage, and one patient had bilateral cerebellar infarct with hemorrhage. All had elevated D-dimer concentrations (>500 ng/ml). Thus, neurological manifestations of COVID-19 infection are not uncommon. Severe neurological complications are either because of direct viral invasion, immunological reaction, or hypoxic metabolic changes as evidenced by Garg et al. [14]. Coagulopathies enhance the risk of cerebral arterial and venous thrombosis in COVID-19. Li et al., in a retrospective study, noted that out of 221 patients, 11 had an acute ischemic stroke [15].

Although acute pancreatitis is a relatively common disease, its occurrence in patients with COVID-19 seems to be rare, and many questions remain unanswered [16]. As of date, there is no evidence for an association between COVID-19 and acute pancreatitis and it is unclear if pancreatitis might be caused by direct viral damage to pancreatic cells or endothelium, or thrombosis and ischemic pancreatitis. In our study, there was one survivor with chronic pancreatitis who had presented with acute illness.

The mortality and severity of complications in COVID-19 patients were significantly associated with liver dysfunction as evidenced by Wu et al. [17]. In our study, the degree of transaminitis (elevated AST) was substantially more in the mortality group compared to the survivor group (p < 0.001).

As in other studies, there was a marked rise in innate immune response, decreased adaptive immune response, an increase of markers of tissue damage, inflammation, and organ failure.

The current study identified many laboratory markers as risk factors of death in adults. Especially, lymphopenia and raised LDH were associated with higher odds of in-hospital death. In addition, elevated levels of neutrophils, monocytes, AST, and serum creatinine were also significantly associated with increased mortality.

The most common causes of death were respiratory failure (84%), ARDS (77%), and cardiac arrest (10%), which were in concordance with Zhou et al. who reported respiratory failure (98%), ARDS (93%), heart failure (52%), and acidosis (30%) as the causes of death among non-survivors [13].

Four patients succumbed to sepsis. Procalcitonin level was below 0.5 ng/ml in 58.2% patients, 0.5-2 ng/ml in 8% patients, 2-10 ng/ml in 3% patients, and more than 10 ng/ml in one deceased patient. Bacterial pathogens were not detected in any of these individuals. These factors conveniently favor that SARS-CoV-2 directly leads to sepsis. This needs further research.

Of the non-survivors, 92% received a combination of low molecular weight heparin, azithromycin, favipiravir, and steroids. Of the non-survivors, 63% were managed with noninvasive ventilation, and 29% of them were mechanically intubated and received tocilizumab. One deceased patient received ECMO therapy. Among survivors, all of them received a combination of azithromycin, favipiravir, and steroids, and 16% of them were managed with noninvasive ventilation.

Our study has several limitations. Due to the retrospective study design, not all laboratory markers were available for all the patients. So the role of important markers like CRP, ferritin, fibrinogen, D-dimer, PT, APTT, serum amylase, and troponin I in contributing to mortality could be underestimated. Although none of the drugs are routinely recommended for COVID-19 pneumonia, a combination of antibiotics, antiviral, and steroids was given to all critically ill patients in the current study. So poor adherence to standard supportive care and inadvertent use of a high dose of steroids also could have contributed to mortality. During the peak of the pandemic, many patients were admitted late in their course of illness, which would have altered the outcome.

## Conclusions

In conclusion, serial monitoring of hematological and coagulation parameters, such as neutrophils, lymphocytes, and D-dimer, and inflammatory and tissue damage markers such as procalcitonin, AST, LDH, serum creatinine, and albumin might provide a reliable and convenient method for classifying and predicting the severity and outcomes of patients with COVID-19.
